# High prevalence of *Sarcocystis* spp. in the Eurasian wolf (*Canis lupus lupus*): Third-generation sequencing resolves mixed infections

**DOI:** 10.1016/j.ijppaw.2025.101140

**Published:** 2025-09-22

**Authors:** Sinah Lückner, Gastón Moré, Iris Marti, Caroline F. Frey, Javier E. Fernandez, Chahrazed Belhout, Walter Basso

**Affiliations:** a Institute of Parasitology, Vetsuisse-Faculty, University of Bern, Länggassstrasse 122, 3012, Bern, Switzerland; b Institute for Fish and Wildlife Health, Vetsuisse-Faculty, University of Bern, Länggassstrasse 122, 3012, Bern, Switzerland; c Division of Molecular Bacterial Epidemiology & Infectious Diseases, Institute of Veterinary Bacteriology, Vetsuisse-Faculty, University of Bern, Länggassstrasse 122, 3012, Bern, Switzerland

**Keywords:** Wolves, Switzerland, Definitive host, Coprological examination, PCR-Sequencing

## Abstract

*Sarcocystis* spp. (Apicomplexa: Coccidia) are obligate heteroxenous protozoa that infect a wide range of host species. Transmission follows a predator-prey cycle involving an intermediate host (IH) and a definitive host (DH). For many species, only IHs have been identified, while DHs remain unknown. DHs can be infected with multiple *Sarcocystis* spp. at the same time, which complicates species identification. We aimed to determine the prevalence and species diversity of *Sarcocystis* infections in free-ranging wolves in Switzerland using both coprological and molecular methods. A further goal was to evaluate the utility of Third-generation sequencing for resolving mixed infections. A total of 87 wolf intestinal content samples were collected between 2017 and 2023 and analyzed coproscopically by a sedimentation-flotation method. *Sarcocystis* oocysts/sporocysts were detected in 76 % (66/87). DNA was obtained from 57/66 positive samples and 55/57 resulted positive in a *Sarcocystis 18S* rRNA screening PCR. Additionally, mitochondrial cytochrome *c* oxidase subunit I (*COI*) gene PCR and a real-time PCR targeting *S. cruzi* were performed. PCR products from conventional PCRs were submitted for Sanger sequencing. Monoinfections were identified in 16 % (9/55) and mixed infections in 84 % (46/55) of the samples. A subset of five samples was analyzed by Third-generation sequencing (Pacific Biosciences) of the *18S* rRNA full-length and *COI* fragment PCR products. BLAST and phylogenetic analysis were used to validate taxonomic classification. Molecular analysis identified nine known *Sarcocystis* species: *S. tenella, S. arieticanis, S. capreolicanis, S. linearis, S. gracilis, S. cruzi, S. capracanis, S. iberica,* and *S. venatoria*. Newly developed pipelines for the Third-generation sequencing data provided high-resolution species-level identification in samples with mixed infections. These findings confirm the Eurasian wolf as natural DH for multiple *Sarcocystis* species for the first time, including *S. linearis*, *S. iberica*, and *S. venatoria*. Further complementary studies on prey species are needed to clarify host-parasite dynamics.

## Highlights

• 76 % intestinal prevalence of *Sarcocystis* spp. in free-ranging wolves in Switzerland.
• Mixed infections detected in 84 % of positive samples.
• High-resolution species identification in mixed infections using NGS.
• Nine known *Sarcocystis* species identified, with up to six species in a single wolf.
• First report of *S. linearis, S. iberica*, and *S. venatoria* in Eurasian wolves as DHs.

## Graphical abstract

**Figure:**
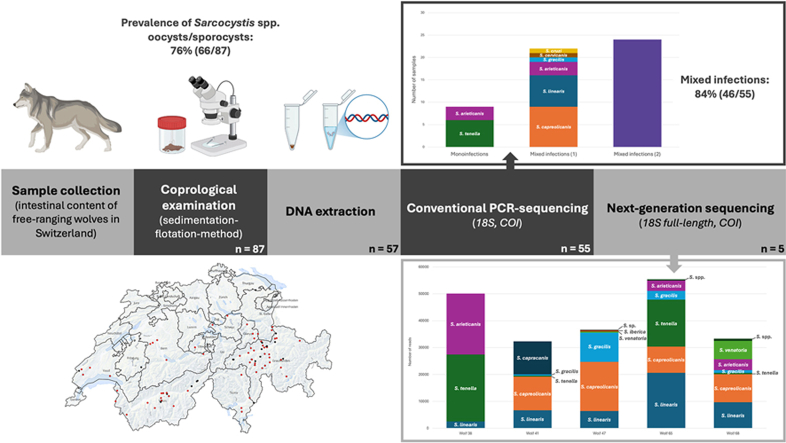

## 1 Introduction

Parasites of the genus *Sarcocystis* (Apicomplexa: Coccidia) are obligate heteroxenous protozoa that infect a wide range of host species, including mammals, birds, reptiles, and fishes. Transmission typically follows a predator-prey cycle: Intermediate hosts (IHs) – commonly herbivores or omnivores, and occasionally carnivores – become infected by ingesting oocysts or sporocysts present in contaminated food or water (Dubey et al., 2016). Within the IH, asexual reproduction occurs, resulting in the development of sarcocysts mainly in striated muscle tissue. Definitive hosts (DHs) – usually carnivores, omnivores, or scavengers (Dubey et al., 2016; Watson et al., 2020) – acquire infection by consuming sarcocyst-containing muscle tissue from IHs. Sexual reproduction and sporulation take place in the intestinal mucosa of the DH, leading to the excretion of infectious oocysts/sporocysts in the feces. In most cases, oocysts/sporocysts are first shed between 7 and 14 days post-infection, with excretion potentially continuing for several months. This can lead to infections with multiple *Sarcocystis* species, causing mixed infections and the simultaneous shedding of morphologically indistinguishable oocysts/sporocysts (Moré et al., 2016; Basso et al., 2020). Host specificity tends to be higher in IHs than in DHs, and in some cases, a single host species may act as both IH and DH for different *Sarcocystis* species. Some species are highly pathogenic to their IHs, especially those transmitted via canids, whereas infections in DHs are generally subclinical (Dubey et al., 2016). To date, over 200 species of *Sarcocystis* have been described, but complete life cycles are only known for a subset. For many species, only the IHs have been identified, but the corresponding DHs remain unknown. While domestic canids are well-established DHs for several species, the role of wild carnivores is not well understood (Boch, 2006; Dubey et al., 2016; Deplazes et al., 2020). We hypothesized that wolves may serve as DHs for several *Sarcocystis* species at the same time, and that some of these species have been described only in IH. These hypotheses gain relevance considering the recent recolonization of Switzerland by the Eurasian wolf (*Canis lupus lupus*). The species was eradicated in Switzerland by the late 19th century following declines in prey species such as red deer, roe deer and wild boar, which intensified human-carnivore conflicts in consequence of increased livestock predations. Legal protection in Italy, along with the recovery of wild ungulate populations in Switzerland, later enabled the Italian wolf population to expand northward, leading to the recolonization of Switzerland in 1995–1996 (*25 Jahre Wolf in der Schweiz*, KORA – *Raubtierökologie und Wildtiermanagement*, https://www.kora.ch/de, accessed June 11, 2025). With the return of this apex predator, new questions have emerged regarding its ecological role in parasite transmission. Their role as a frequent DH could already be demonstrated for various cestode species, particularly those involving ruminants as IHs (Schneider et al., 2025). Predator-prey interactions of wolves highlight their importance in understanding life cycles of parasites, particularly those affecting ruminant hosts.

The prevalence of *Sarcocystis* spp. in free-ranging wolf populations has been assessed in several geographic regions (Table 1), but some studies investigating the wolf as DH have been inconclusive. Early research mainly relied on experimental infections (with complementary coprological evidence) conducted before the development of molecular diagnostics (Dubey et al., 2016). More recent studies have exclusively used molecular methods, based on fecal DNA extraction and PCR amplification, without complementary coprological evidence (Lesniak et al., 2017, 2018). To date, only one study has employed both coprological and molecular methods in combination (Gupta et al., 2024b). As a result, for several *Sarcocystis* species, the wolf as natural DH remains unconfirmed and requires further validation (Table 2). In parallel, recent discoveries have revealed that wolves can also serve as IHs for *S. arctica/S. caninum* (Calero-Bernal et al., 2016; Gupta et al., 2024a; Juozaitytė-Ngugu et al., 2024) with corvids (Juozaitytė-Ngugu et al., 2021) or raptors (Máca and González-Solís, 2022; Juozaitytė-Ngugu et al., 2025) as potential DHs, and for *S. svanai* (Gupta et al., 2024a; Juozaitytė-Ngugu et al., 2024; Dubey et al., 2025), for which the DHs remain unknown. This highlights the complexity of the *Sarcocystis* spp. epidemiology.

**Table 1:** Prevalence of intestinal infections with *Sarcocystis* spp. in free-ranging wolf populations in several geographic regions based on coprology.

Country (region)	Prevalence	Reference
Canada (Newfoundland and Labrador)	15/15 (100 %)	Khan and Evans (2006)
Canada (Manitoba):	Years: 2001–2003: 120/320 (37.5 %)	Stronen et al. (2011)
-Riding Mountain National Park (RMNP)	Years: 2003–2005: 58/159 (36.5 %)	
-Duck Mountain Provincial Park and Forest (DMPPF)	46/122 (37.7 %)	
Canada (British Columbia)	681/1558 (43.7 %)	Bryan et al. (2012)
Croatia (Gorski Kotar)	76/400 (19.1 %)	Hermosilla et al. (2017)
Italy (Abruzzo): Lazio e Molise National Park (PNALM)	9/88 (10.2 %)	Molnar et al. (2019)
France (Mercantour): Mercantour National Park (PNM)	3/68 (4.4 %)	
USA (Yellowstone): Yellowstone National Park (YNP)	63/186 (33.9 %)

**Table 2:** Confirmed or suspected *Sarcocystis* spp. identified in wolves as DHs based on different diagnostic approaches, and the known corresponding IHs.

Method	*Sarcocystis* species	Corresponding IHs	Reference
Not reported	*S. miescheriana*	Pig (*Sus domesticus*)^c^	Dubey et al. (2016)^m^
		Wild boar (*Sus scrofa*)^c^	
	*S. baibacinacanis*	Gray marmot (*Marmota baibacina*)^c^	
Experimental infection (with complementary coprological evidence)	*S. cruzi*	Cattle (*Bos taurus*)^c^	
		American bison (*Bison bison*)^c^	
		European bison (*Bison bonasus*)^c^	
		Banteng (*Bos javanicus*)^c^	
	*S. odocoileocanis*	White-tailed deer (*Odocoileus virginianus*)^c^	
Molecular examination of feces	*S. arieticanis*	Sheep (*Ovis aries*)^c^	(Lesniak et al., 2017, 2018)
		European mouflon (*Ovis aries musimon*)^c^	
		Goat (*Capra hircus*)^j^	
	*S. capracanis*	Goat (*Capra hircus*)^c^	
		Sheep (*Ovis aries*)^j^	
		Barbary sheep (*Ammotragus lervia*)^h^	
		Alpine ibex (*Capra ibex*)^l^	
		European mouflon (*Ovis aries musimon*)^i^	
	*S. capreolicanis*	Roe deer (*Capreolus capreolus*)^c^	
	*S. cruzi*	See above	
	*S. gracilis*	Roe deer (*Capreolus capreolus*)^c^	
		European fallow deer (*Dama dama*)^f^	
	*S. grueneri*	Reindeer (*Rangifer tarandus*)^c^	
		Wapiti (*Cervus canadensis*)^c^	
		Water deer (*Hydropotes inermis*)^e^	
	*S. hjorti*	Red deer (*Cervus elaphus*)^c^	
		Moose (*Alces alces*)^c^	
		Sika deer (*Cervus nippon*)^k^	
	*S. miescheriana*	See above	
	*S. rangi*	Reindeer (*Rangifer tarandus*)^c^	
	*S. taeniata*	Moose (*Alces alces*)^c^	
		Sika deer (*Cervus nippon*)^d^	
	*S. tenella*	Sheep (*Ovis aries*)^c^	
		European mouflon (*Ovis aries musimon*)^c^	
		Goat (*Capra hircus*)^j^	
		Barbary sheep (*Ammotragus lervia*)^h^	
		Tatra chamois (*Rupicapra rupicapra tatrica*)^a^	
	*S. bovini*	Cattle (*Bos taurus*)^c^	
	*S. elongata*	Red deer (*Cervus elaphus*)^c^	
		Sika deer (*Cervus nippon*)^k^	
	*S. silva*	Roe deer (*Capreolus capreolus*)^c^	
		Moose (*Alces alces*)^c^	
		Sika deer (*Cervus nippon*)^k^	
		Red deer (*Cervus elaphus*)^g^	
	*S. tarandi*	Reindeer (*Rangifer tarandus*)^c^	
		Red deer (*Cervus elaphus*)^c^	
	*S. truncata*	Red deer (*Cervus elaphus*)^c^	
		Sika deer (*Cervus nippon*)^k^	
Coprological and molecular examination	*S. cruzi*	See above	Gupta et al. (2024b)
	*S. mehlhorni*	Black-tailed deer (*Odocoileus hemionus columbianus*)^b^	
	*S. wenzeli*	Chicken (*Gallus gallus domesticus*)^c^	

a (Kolenda et al., 2015).

b (Calero-Bernal et al., 2015).

c (Dubey et al., 2016).

d (Prakas et al., 2016).

e (Kim et al., 2018).

f (Cabaj et al., 2020).

g (Basso et al., 2020).

h (Delgado-de Las Cuevas et al., 2021).

i (Prakas et al., 2021).

j (Marandykina-Prakienė et al., 2023).

k (Prakas et al., 2023).

l (Rudaitytė-Lukošienė et al., 2025).

m Original references in (Dubey et al., 2016).

Identifying different *Sarcocystis* species in mixed infections in DHs is challenging because released parasite stages are morphologically indistinguishable, and conventional PCR-sequencing methods lack resolution (Juozaitytė-Ngugu et al., 2021). To overcome these limitations, advanced molecular techniques – particularly next-generation sequencing (NGS) – are becoming increasingly attractive. Third-generation sequencing technologies, such as Pacific Biosciences (PacBio), enable sequencing with reported accuracies of up to 99.9 % (Callahan et al., 2019). These platforms offer great potential for resolving mixed infections and improving our understanding of host-parasite relationships in wildlife systems.

This study aimed to: (1) determine the prevalence of intestinal *Sarcocystis* spp. infection in free-ranging wolves in Switzerland; (2) identify the species involved using both coprological and molecular methods; and (3) critically assess which species can be reliably attributed to wolves as DHs. A further goal was to evaluate the performance of Third-generation sequencing (PacBio) for resolving mixed *Sarcocystis* infections.

## 2 Material and methods

### 2.1 Sample collection

Between May 2017 and April 2023, a total of 87 intestinal content samples were collected from free-ranging wolves in Switzerland at necropsy. Carcasses of all wolves that were either found dead or legally culled by the hunting administration were submitted to the Institute of Fish and Wildlife Health, University of Bern, for post-mortem examination and collection of baseline health data as a part of a long-term monitoring program (*Konzept Wolf Schweiz*, CHWOLF, https://chwolf.org, accessed July 21, 2025) (Schneider et al., 2025). Intestinal content samples were subsequently forwarded to the Institute of Parasitology, University of Bern, for further coprological examination. For biosafety reasons, all samples were frozen at −80 °C for at least three days to inactivate any potentially present *Echinococcus* spp. eggs prior to processing. The sex, estimated age, and origin of the wolves were recorded.

### 2.2 Coprological examination

All samples (n = 87) were analyzed using a sedimentation-flotation method employing a 44 % zinc chloride solution (specific gravity: 1.3) as described previously (Deplazes et al., 2020). The samples were examined microscopically at 200x and 400x magnification for the presence of *Sarcocystis* spp. oocysts/sporocysts. A subset of samples containing oocysts/sporocysts (n = 57) was available and with enough material for molecular analysis and subsequently included in this study. In samples containing a high amount of oocysts/sporocysts, the sediment was directly transferred into a 2 ml tube. In samples with a low amount of oocysts/sporocysts, an additional flotation method using a concentrated sucrose solution (specific gravity: 1.3) was performed to enhance the concentration of parasitic elements (Deplazes et al., 2020). In these cases, material from the top of the flotation fluid was transferred to a 2 ml tube using a wire loop, totalizing about 150 μl. The collected material was conserved at −20 °C until DNA extraction was performed.

### 2.3 Molecular examination

#### 2.3.1 DNA extraction

DNA was extracted from ∼150 mg (or μl) of intestinal content sediment or flotation fluid using the Quick-DNA Fecal/Soil Microbe Miniprep Kit (Zymo Research, USA), according to the manufacturer's instructions, performing the mechanical rupture using the TissueLyser II (QIAGEN, USA) with 30 movements/sec for 10 min.

#### 2.3.2 Polymerase chain reactions (PCRs) and Sanger sequencing

Different conventional PCRs were performed targeting the small subunit ribosomal RNA gene (*18S*) and a fragment of the mitochondrial cytochrome *c* oxidase subunit I (*COI*) gene of *Sarcocystis* spp. Additionally, a specific real time PCR targeting a fragment of the *18S* of *S. cruzi* was also performed.

*18S* (*screening*) PCR: A conventional PCR targeting an ∼700 bp fragment of the *Sarcocystis* spp. *18S* was performed using the primers SarcoF and SarcoR (Moré et al., 2011). Each PCR mixture contained 12.5 μL QIAGEN Multiplex Master Mix, 9.5 μL ddH_2_O, 0.25 μL of each primer (100 μM solution), and 2.5 μL of extracted DNA, for a final volume of 25 μL. A positive control (*S. miescheriana*) and a non-template control (NTC; RNase-free water) were included in each run. Amplification was performed in a thermal cycler (GeneAmp PCR System 9700, Applied Biosystems) under the following conditions: 94 °C for 15 min; 40 cycles of 94 °C for 40 s, 59 °C for 30 s, and 72 °C for 1 min; followed by a final extension at 72 °C for 10 min and a hold at 4 °C. PCR products were separated by electrophoresis on 1.5 % agarose gels stained with ethidium bromide, and were visualized and photographed using a UV imaging system (E-Box, Vilber, France). Samples that tested negative were re-amplified once for confirmation.

*COI* PCR: A conventional PCR targeting an ∼1000–1100 bp fragment of the *Sarcocystis* spp. *COI* was performed using the primers SF1 and SR9 (Gjerde, 2013). Each PCR mixture contained 12.5 μL QIAGEN Multiplex Master Mix, 9.2 μL ddH_2_O, 0.15 μL of each primer (100 μM solution), and 3 μL of extracted DNA, for a final volume of 25 μL. A positive control (*S. miescheriana*) and an NTC (RNase-free water) were included in each run. Amplification was performed in the same thermal cycler under the following conditions: 95 °C for 15 min; 45 cycles of 94 °C for 30 s, 55 °C for 30 s, and 72 °C for 90 s; followed by a final extension at 72 °C for 10 min and a hold at 4 °C. Subsequent steps were performed as described for the *18S* (*screening*) PCR.

Real-time PCR: A real-time PCR targeting an ∼166 bp fragment of the *18S* rRNA gene of *Sarcocystis* spp. was performed using the primers SarcoRTF and SarcoRTR, along with a specific *S. cruzi* TaqMan probe (Moré et al., 2013). Each PCR mixture contained 10 μL TaqMix (SensiFAST™ Probe NO-ROX Kit; Bioline Meridian Lifescience, Memphis, TN, USA), 7.14 μL ddH_2_O, 0.12 μL of each primer (100 μM solution), 0.12 μL of the TaqMan probe (10 μM solution), and 2.5 μL of extracted DNA, for a final volume of 20 μL. A positive control (*S. cruzi*) and an NTC (RNase-free water) were included in each run. Amplification was performed in a thermal cycler (CFX96 Bio-Rad) under the following conditions: 95 °C for 5 min; 40 cycles of 95 °C for 15 s and 62 °C for 40 s. Real-time amplifications were analyzed using the CFX Manager Software (Bio-Rad Laboratories GmbH, Germany) under the Texas Red channel (Moré et al., 2013).

The amplified DNA bands from the two conventional PCR assays were excised from the agarose gel and purified using the Zymoclean Gel DNA Recovery Kit (Zymo Research, USA), according to the manufacturer's instructions. The concentration and purity of the recovered DNA were assessed using a spectrophotometer (NanoDrop One, Thermo Scientific). Purified PCR products were submitted to Microsynth AG (Balgach, Switzerland; https://srvweb.microsynth.ch) for bidirectional Sanger sequencing, using the same primers as in the corresponding PCR assays. Forward and reverse sequences were aligned using Geneious Prime Software (https://www.geneious.com) to generate consensus sequences. These were compared with reference sequences in the GenBank databank (https://www.ncbi.nlm.nih.gov) using BLAST. Sequences with ≥99 % identity were assigned to the corresponding *Sarcocystis* species. Samples resulting in short *Sarcocystis* sequences, or showing chromatograms with double/superimposed peaks, from which a consensus could not be defined, were assumed as “mixed infections”.

#### 2.3.3 Additional PCRs and third-generation long-read sequencing

*18S* (*full-length*) PCR: A conventional PCR targeting the complete ∼1900 bp small subunit ribosomal RNA gene of *Sarcocystis* spp. was performed on five samples (wolves 38, 41, 47, 65, and 68) previously identified as “mixed infections”, using the primers ERIB1 and Primer B proceeding essentially as described (Moré et al., 2013). Briefly, each PCR mixture contained 12.5 μL QIAGEN Multiplex Master Mix, 9.5 μL ddH_2_O, 0.25 μL of each primer (100 μM solution), and 2.5 μL of extracted DNA, for a final volume of 25 μL. A positive control (*S. miescheriana*) and an NTC (RNase-free water) were included in each run. Amplification was performed in the same thermal cycler as the other conventional PCRs under the following conditions: 94 °C for 15 min; 40 cycles of 94 °C for 40 s, 58 °C for 1 min, and 72 °C for 2 min; followed by a final extension at 72 °C for 5 min and a hold at 4 °C. PCR products were separated using the QIAxcel Connect Capillary Gel Electrophoresis System (QIAGEN, Germany).

Third-generation long-read sequencing (PacBio sequencing): Non-purified PCR products (purification was included in the service) of the *18S* (*full-length*) PCR and the *COI* PCR (was repeated as previously described (Section 2.3.2)) from the five above mentioned samples, were submitted to the Next Generation Sequencing Platform, University of Bern. The resulting amplicons were evaluated for quantity, size and purity using a Qubit 4.0 fluorometer with the Qubit dsDNA BR and HS Assay Kit (Thermo Fisher Scientific), and an Advanced Analytical FEMTO Pulse instrument Agilent with a HS NGS Fragment Kit (Agilent). Multiplexed SMRTbell libraries were prepared using the HiFi plex prep kit 96 according to manufacturer instructions (https://www.pacb.com/documentation/procedure-checklist-preparing-multiplexed-whole-genome-and-amplicon-libraries-using-the-hifi-plex-prep-kit-96/). Briefly, amplicons initially underwent the end repair and A-tailing step followed by Adapter ligation and termination. Thereafter, the libraries were equimolar pooled, and the library pool was also assessed for quantity, purity and fragment size as outlined above. The library pool was combined with other libraries and prepared for HiFi sequencing using a PacBio Revio® instrument with a 25M SMRT cell. Instructions in SMRT Link Sample Setup were followed to prepare the SMRTbell library for sequencing (PacBio SMRT Link v25). Shortly, the PacBio standard sequencing primer was annealed to the SMRTbell libraries, next the Revio DNA Polymerase was bound, and the polymerase bound complex was bead-based purified. Finally, the Revio sequencing control DNA was diluted and spiked into the complex prior to pipetting onto the thawed Revio SPRQ sequencing plate (Revio SPRQ polymerase kit + cleanup beads, PacBio). The Revio deck was setup as directed from the SMRTLink software and included laying out tips, sequencing plates and Revio SMRT Cell trays containing 4 x SMRT cell 25M into their designated locations. The library pool was loaded at an on-plate concentration of 400 pM using adaptive loading. SMRT sequencing was performed on the Revio with a 24 h movie time. Demultiplexing was performed post-run in SMRTLink.

#### 2.3.4 Bioinformatics

Raw sequencing data were initially filtered based on primer presence and sequence length (*18S*: 1824–1890 bp, *COI*: 950–1150 bp). Subsequent processing was performed within the QIIME 2 environment using DADA2, which included primer removal, length trimming (*18S*: 1801–1870 bp, *COI*: 1000–1100 bp), and denoising to generate amplicon sequence variants (ASVs). For taxonomic classification, the SILVA 138.2 database was used for *18S* reads, while a custom classifier based on curated reference sequences was applied for *COI* reads. For both datasets, sequences assigned to the genus *Sarcocystis* were filtered and retained for further phylogenetic analysis. A threshold of 5 reads was set up to consider a sample as positive for a given *Sarcocystis* sp. ASV. For the *COI* dataset, sequences were further clustered into operational taxonomic units (OTUs) at a 97.5 % identity threshold to facilitate downstream analyses of sequences with higher variability. Representative ASVs or OTUs were aligned with a manually selected set of reference sequences using the Geneious Prime Software (https://www.geneious.com). Multiple sequence alignments were used to construct phylogenetic trees using the MrBayes Plugin from Geneious Prime Software, applying the HYK85 substitution model with a *Toxoplasma gondii* sequence (*18S*: TGU03070 from RH strain, *COI*: KM657810) as the outgroup. Taxonomic assignments were validated using both BLAST and rooted phylogenetic trees.

### 2.4 Statistical methods

A chi-square (χ^2^) test was performed using the online tool WinEpi (http://www.winepi.net/) with a 95 % confidence level to compare *Sarcocystis* spp. positivity rates by sex, age group, and origin. Differences were considered statistically significant at *p* < 0.05.

## 3 Results

### 3.1 Coprological examination

**Fig. 1:**
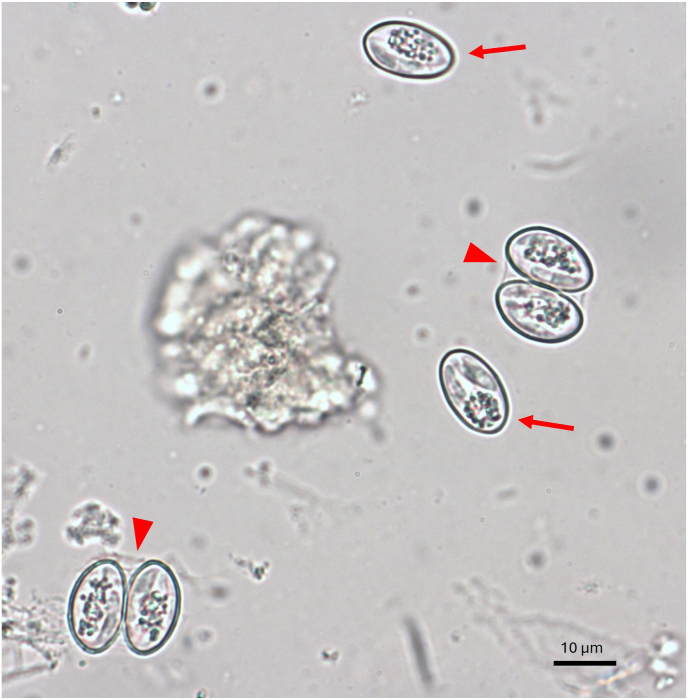
Photomicrography of *Sarcocystis* spp. oocysts (arrow heads) and free sporocysts (arrows) identified in the intestinal content of a wolf (wolf 3) by zinc chloride flotation.

**Fig. 2:**
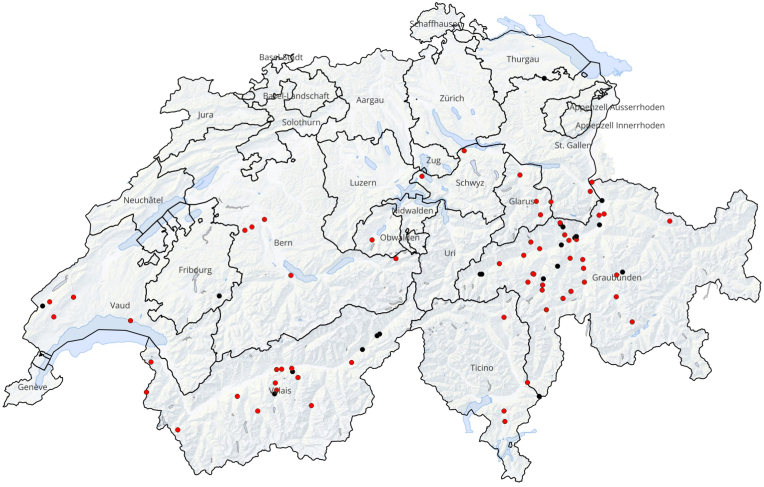
Geographic distribution of the studied free-ranging wolves in Switzerland created with the Quantum Geographic Information System (QGIS, https://www.qgis.org). Red dots indicate samples positive for *Sarcocystis* spp. by coprology; black dots indicate negative samples. It is important to note that the points shown only represent carcass recovery sites and do not reflect the whole territorial range of the wolves.

The examined wolf samples included 58 males and 29 females. 39 samples were obtained from wolves younger than one year, while 48 samples originated from wolves older than one year. Out of a total of 87 samples analyzed, relatively well-preserved oocysts/sporocysts of *Sarcocystis* spp. (Fig. 1) were detected in 66 samples, yielding an overall prevalence of 76 % in free-ranging wolves in Switzerland. Statistical analysis showed no significant difference in *Sarcocystis* spp. positivity between males (74 %; 43/58) and females (79 %; 23/29) (*p* = 0.5933), nor between the age groups of <1 year (77 %; 30/39) or > 1 year (75 %; 36/48) (*p* = 0.8368). Most of the samples came from animals of the cantons of Grisons (72 %; 31/43) and Valais (74 %; 14/19). The overall *Sarcocystis* spp. positivity among samples from other cantons (Bern, Ticino, Vaud, Glarus, Schwyz, Fribourg, Obwalden, and St. Gallen) considered together, was 84 % (21/25). No statistically significant difference was observed in positivity among wolves from Grisons, Valais, and the other cantons (*p* = 0.5217). Fig. 2 illustrates the geographic distribution of the sampled free-ranging wolves in Switzerland. Of the 66 positive samples in coprology, 57 samples could be archived for further molecular analysis.

### 3.2 Molecular examination

#### 3.2.1 Polymerase chain reactions (PCRs) and Sanger sequencing

*18S* (*screening*) PCR: *Sarcocystis* DNA was successfully amplified in 55 of the 57 samples analyzed. Subsequent sequencing revealed monoinfections in 16 % (9/55) of the samples, consisting of *S. tenella* (6/9) and *S. arieticanis* (3/9), respectively (Table 3). The remaining 84 % (46/55) exhibited mixed infections.

**Table 3:** *Sarcocystis* spp. *18S* (*screening*) PCR and Sanger sequencing: List of the monoinfections of the analyzed free-ranging wolves in Switzerland.

GenBank accession no.	Wolf ID	*Sarcocystis* species	BLAST identity in % (sequence length)^c^	Reference sequences
**PV993815**	Wolf 13	*S*. *tenella*	100 (647 bp)^d^	KP263759.1, PQ182258.1, MW832470.1, among others
a	Wolf 24, 40, 54, 61, 63		100 (647-589 bp)	
**PV993816**	Wolf 62	*S*. *arieticanis*	100 (635 bp)	PQ538540.1, MF039330.1, MK420017.1
b	Wolf 44, 64		100 (590-584 bp)	

a Sequences identical to **PV993815** and not submitted to GenBank.

b Sequences identical to **PV993816** and not submitted to GenBank.

c All samples showed 100 % coverage with the reference sequences in BLAST.

d Length from primer to primer, with trimmed primers.

*COI* PCR (mixed infections, n = 46)*:* Two samples were excluded from sequencing due to the presence of several unspecific bands, leaving 44 samples for analysis. Among these, 48 % (21/44) contained mixed infections, with short consensus or fragments from one of the single sequences with high identity to defined *Sarcocystis* species: *S. capreolicanis* (9/21), *S. linearis* (7/21), *S. arieticanis* (3/21), *S. gracilis* (1/21), and *S. cervicanis* (1/21) (Table 4). The remaining 52 % (23/44) represented mixed infections with unidentifiable *Sarcocystis* species sequences.

**Table 4:** *Sarcocystis* spp. *COI* PCR and Sanger sequencing: List of the mixed infections with sequence fragments with high identity to defined *Sarcocystis* species of the analyzed free-ranging wolves in Switzerland.

GenBank accession no.	Wolf ID	*Sarcocystis* species	BLAST identity in % (sequence length)^±^	Reference sequences
**PX000702**	Wolf 18	*S*. *capreolicanis*	100 (1026 bp)	KY018943.1
**PX000703**	Wolf 46		100 (1031 bp)	
NS	Wolf 14		99.31 (1009 bp)	KY018944.1
NS	Wolf 15, 30, 75, 80		100 (1018-573 bp)	KY018943.1, MN339281.1
NS	Wolf 33		100 (318 bp)	PP935191.1, KY018943.1, MN339281.1
NS	Wolf 87		100 (293 bp)	PP935191.1, KY018943.1, MN339281.1, among others
**PX000704**	Wolf 49	*S*. *linearis*	99.70 (1013 bp)	MT070669.1
NS	Wolf 3		99.54 (432 bp)	KY018971.1, PP935196.1, MT070667.1
NS	Wolf 17		99.43 (882 bp)	MT070665.1, KY973297.1
NS	Wolf 45		99.04 (209 bp)	KY018971.1, PP935196.1, MT070667.1, among others
NS	Wolf 52		99.67 (599 bp)	MN339324.1
NS	Wolf 71, 74		100 (288-278 bp)	KY018971.1, PP935196.1, MT070667.1, among others
NS	Wolf 37	*S*. *arieticanis*	99.37 (318 bp)	PQ165949.1, MK419975.1
NS	Wolf 38		100 (179 bp)	MF039324.1
NS	Wolf 57		100 (365 bp)	PQ165949.1, ON858962.1
**PX000705**	Wolf 35	*S*. *gracilis*	100 (1025 bp)	KY018947.1
NS	Wolf 42	*S*. *cervicanis*	100 (299 bp)	KY973295.1

NS: Not submitted to GenBank because an assembled consensus was not achieved,±All samples showed 100 % coverage with the reference sequences in BLAST.

Real-time PCR (mixed infections, n = 46)*:* A mixed infection involving *S. cruzi* was detected in one of the samples (2 %; 1/46).

**Fig. 3:**
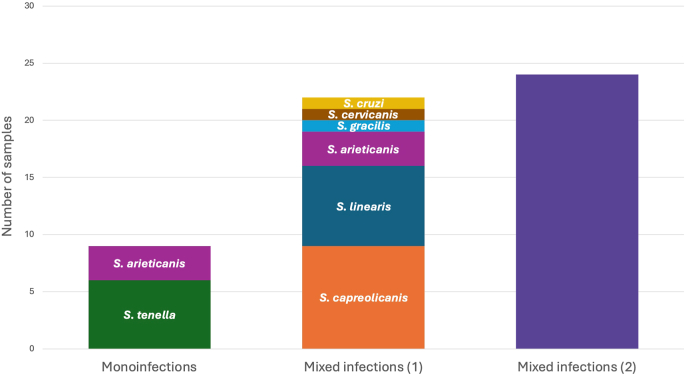
Graphical representation of the combined results of *18S* (*screening*) PCR, *COI* PCR, real-time PCR, and Sanger sequencing. The columns show the number of samples with monoinfections, as well as two types of mixed infections: (1) Mixed infections with sequence fragments with high identity to defined *Sarcocystis* spp. (2) Mixed infections with undetermined *Sarcocystis* spp. sequences.

Among the 55 successfully amplified samples, considering all together *18S* (*screening*) PCR, *COI* PCR, real-time PCR, and Sanger sequencing results, monoinfections were detected in 16 % (9/55), while mixed infections were identified in 84 % (46/55) of the samples. Overall*,* in mixed infections*,* sequence fragments with high identity to defined *Sarcocystis* spp. were identified in 40 % (22/55), whereas in 44 % (24/55) of the samples the *Sarcocystis* species could not be determined (Fig. 3).

#### 3.2.2 Additional PCRs and NGS

**Fig. 4:**
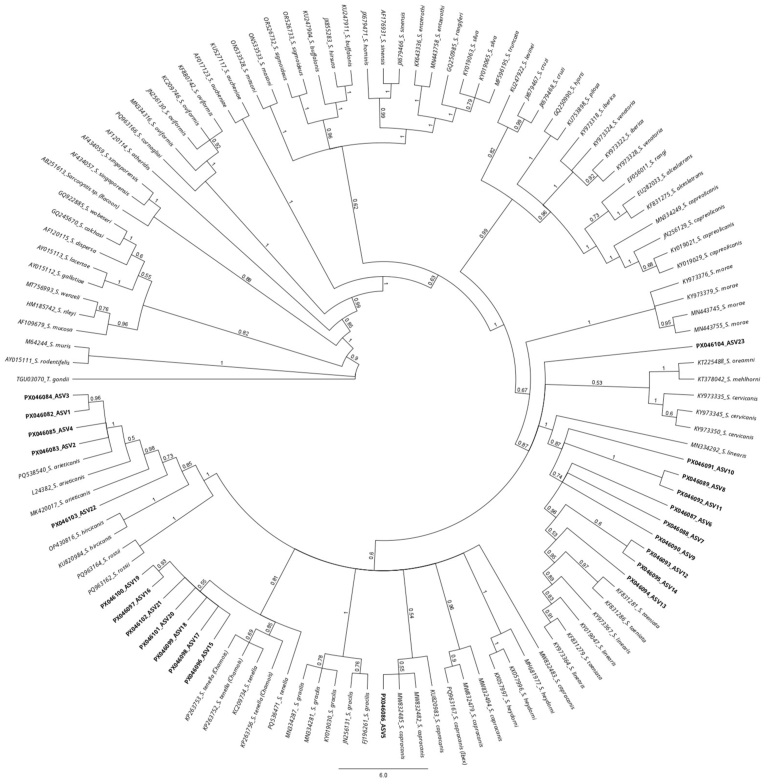
Phylogenetic tree based on a multi-alignment of the 23 ASVs for *18S* full-length obtained in the present study (**in bold**) along with *Sarcocystis* spp. *18S* reference sequences. The figure represents the posterior output of the MrBayes Plugin of Geneious Prime Software. A *18S* sequence of *T. gondii* (TGU03070 from RH strain) was used as the outgroup.

**Fig. 5:**
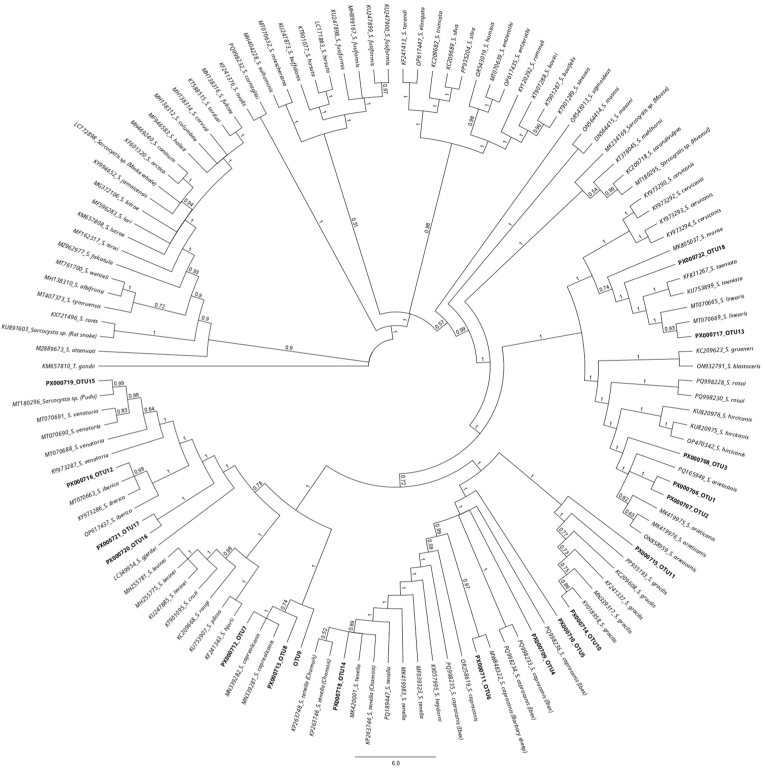
Phylogenetic tree based on a multi-alignment of the 18 OTUs for *COI* obtained in the present study (**in bold**) along with *Sarcocystis* spp. *COI* reference sequences. The figure represents the posterior output of the MrBayes Plugin of Geneious Prime Software. A *COI* sequence of *T. gondii* (KM657810) was used as the outgroup.

High-throughput sequencing produced over 1.5 million circular consensus sequencing (CCS) reads across the five analyzed samples. Out all the raw reads obtained for *COI* (669,247), 24.7 % (165,118) were assigned to *Sarcocystis* spp., while 4.9 % (42,565) from the total reads of the complete *18S* (865,912) were assigned to *Sarcocystis* spp. The newly developed bioinformatics pipelines (**Supplementary data 3**) identified 23 ASVs for *18S* (Table 5, Fig. 4) and 173 ASVs clustered into 18 OTUs for *COI* (Table 6, Fig. 5) which were further analyzed.

**Table 5:** *Sarcocystis* spp. *18S* (*full-length*) PCR and PacBio sequencing: List of the 23 ASVs of the analyzed free-ranging wolves in Switzerland and the corresponding number of reads per ASV and sample.

GenBank accession no.	ASV ID	*Sarcocystis* species	Wolf 38	Wolf 41	Wolf 47	Wolf 65	Wolf 68
**PX046082**	ASV 1	*S*. *arieticanis*	6254	0	0	0	0
**PX046083**	ASV 2		1194	0	0	6	1099
**PX046084**	ASV 3		11	0	0	1428	0
**PX046085**	ASV 4		0	0	0	457	0
**PX046086**	ASV 5	*S*. *capracanis-*like^a^	0	217	0	0	0
**PX046087**	ASV 6	*S*. *linearis*	0	0	0	2521	0
**PX046088**	ASV 7		0	0	0	0	1423
**PX046089**	ASV 8		0	0	0	1261	0
**PX046090**	ASV 9		0	0	0	1225	0
**PX046091**	ASV 10		0	0	0	898	0
**PX046092**	ASV 11		0	0	0	261	0
**PX046093**	ASV 12	*S*. *linearis-*like^a^	0	0	0	1305	0
**PX046094**	ASV 13		0	0	0	692	0
**PX046095**	ASV 14		0	0	0	481	0
**PX046096**	ASV 15	*S*. *tenella*	6698	0	0	7730	0
**PX046097**	ASV 16		3116	0	0	3326	0
**PX046098**	ASV 17		0	0	0	137	0
**PX046099**	ASV 18		0	0	0	22	0
**PX046100**	ASV 19		0	0	0	17	0
**PX046101**	ASV 20		0	0	0	5	0
**PX046102**	ASV 21	*S*. *tenella-*like^a^	205	0	0	0	0
**PX046103**	ASV 22	*Sarcocystis* sp.∗^a^	0	0	0	179	0
**PX046104**	ASV 23	*Sarcocystis* sp.^b^	0	0	28	216	153
		Total reads per sample	17478	217	28	22167	2675

a In phylogenetic tree in a branch most closely related to the named species, ∗∗ In phylogenetic tree in a branch with more than one species.

b In phylogenetic tree isolated with no affiliation to any other species.

**Table 6:** *Sarcocystis* spp. *COI* PCR and PacBio sequencing: List of the 18 OTUs of the analyzed free-ranging wolves in Switzerland and the corresponding number of reads per OTU and sample.

GenBank accession no.	OTU ID	*Sarcocystis* species	Wolf 38	Wolf 41	Wolf 47	Wolf 65	Wolf 68
**PX000706**	OTU 1	*S*. *arieticanis*	14723	0	0	1947	2946
**PX000707**	OTU 2		489	0	0	0	0
**PX000708**	OTU 3		0	0	0	49	0
**PX000709**	OTU 4	*S*. *capracanis*	0	11747	0	0	0
**PX000710**	OTU 5		0	165	0	0	0
**PX000711**	OTU 6		0	109	0	0	0
**PX000712**	OTU 7	*S*. *capreolicanis*	0	11990	17664	9762	10426
**PX000713**	OTU 8		0	608	437	0	0
NR	OTU 9		0	0	212	0	0
**PX000714**	OTU 10	*S*. *gracilis*	0	417	10270	3236	1094
**PX000715**	OTU 11		0	0	582	0	0
**PX000716**	OTU 12	*S*. *iberica*	0	0	626	0	0
**PX000717**	OTU 13	*S*. *linearis*	2526	6641	6406	11969	8249
**PX000718**	OTU 14	*S*. *tenella*	14893	405	0	6240	489
**PX000719**	OTU 15	*S*. *venatoria*	0	0	402	0	6825
**PX000720**	OTU 16	*Sarcocystis* sp.^a^	0	0	0	0	425
**PX000721**	OTU 17		0	0	0	0	93
**PX000722**	OTU 18		0	0	0	0	56
		Total reads per sample	32631	32082	36599	33203	30603

NR: Non reportable. The sequence has good quality but, in the translation contains a codon stop, making it not reportable in the GenBank.

a In phylogenetic tree in a branch with more than one species.

The *Sarcocystis capracanis*-like ASV (**PX046086**) shared a 99.89 % identity with *S. capracanis* (MW832485.1) and 99.46 % with *S. tenella* (KC209734.1) in BLAST analysis. Phylogenetically, it clustered within a branch containing *S. capracanis* sequences. Taking into account the short fragment (used in the *18S* screening PCR) of the *18S* sequence it showed 100 % identity exclusively with *S. capracanis* (L76472.1, PQ288429.2). Three *Sarcocystis linearis*-like ASVs (**PX046093**, **PX046094**, **PX046095**) displayed high BLAST identities of 99.73 % (KY973357.1), 99.62 % (MN334298.1), and 99.67 % (KY973367.1) with *S. linearis*. These ASVs grouped within a phylogenetic branch containing *S. linearis* and *S. taeniata*. In case of (**PX046094**), the short fragment of the *18S* sequence showed 100 % identity only with *S. linearis* (KY973367.1, MN334290.1). The *Sarcocystis tenella*-like ASV (**PX046102**) had a top BLAST match of 95.37 % with *S. tenella* (KC209737.1) and clustered within a phylogenetic branch alongside *S. tenella* with a lower support. A *Sarcocystis* sp. ASV (**PX046103**) showed a maximum BLAST identity of 95.07 % with *S. hircicanis* (KU820985.1) and was placed in a clade containing *S. hircicanis*, *S. arieticanis*, and *S. rossi*. Another *Sarcocystis* sp. ASV (**PX046104**) demonstrated a maximum BLAST identity of 91.11 % with *S. cruzi* (OR553290.1) and was phylogenetically isolated, forming a distinct lineage with no clear affiliation to known *Sarcocystis* species.

A *Sarcocystis* sp. OTU (**PX000720**) exhibited a maximum BLAST identity of 96.80 % with a *Sarcocystis* sp. from *Pudu puda* (MT180296.1) and clustered phylogenetically with *S. venatoria* and *S. iberica.* Another *Sarcocystis* sp. OTU (**PX000721**) shared 94.61 % identity with *S. venatoria* (MT070688.1) grouped within a clade comprising *S. venatoria* and *S. iberica.* The last *Sarcocystis* sp. OTU (**PX000722**) exhibited 95.76 % identity with *S. linearis* (MK234164.1) and clustered within a branch comprising *S. linearis* and *S. taeniata.*

**Fig. 6:**
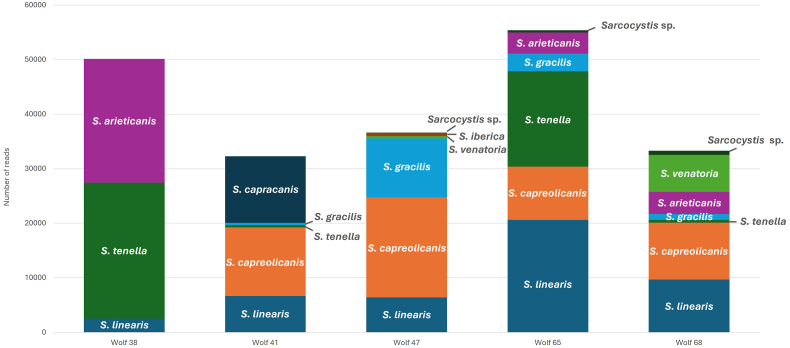
Graphical representation of the identified *Sarcocystis* species per wolf by combining the PacBio sequencing results of the *18S* (*full-length*) PCR and *COI* PCR. Note that at least between three and six known *Sarcocystis* species were detected in a single wolf.

Based on the combined application of all diagnostic methods, a total of nine known *Sarcocystis* species were certainly identified: *S. tenella, S. arieticanis, S. capreolicanis, S. linearis, S. gracilis, S. cruzi, S. capracanis, S. iberica,* and *S. venatoria* (Fig. 6)*.* Only a short fragment *COI* sequence of *S. cervicanis* was identified (Table 4) and not further considered as a properly identified species.

## 4 Discussion

Although *Sarcocystis* spp. are common parasites in a variety of host species, relatively little is known about their occurrence in wolves. This study represents the first investigation of *Sarcocystis* infections in wolves as DHs from Switzerland. We found a prevalence of 76 %, which is considerably higher than reported rates in studies with similar sample sizes from other regions, such as Canada (36.5–43.7 %) (Stronen et al., 2011; Bryan et al., 2012) and Croatia (19.1 %) (Hermosilla et al., 2017). Possibly, the use of intestinal content samples in our study, or the detection method employed, allowed a higher sensitivity in oocyst/sporocyst detection than that reported in other studies. Moreover, since we observed intact oocysts/sporocysts without signs of degradation, we assume their presence due to an active infection in wolves and not due to a passage after digestion of intestinal tissues of another predator species. Positivity did not differ markedly between sexes (males: 74 %; 43/58, females: 79 %; 23/29) or age groups (<1 year: 77 %; 30/39, >1 year: 75 %; 36/48), a pattern consistent with the social structure of wolf packs, where all members typically feed on the same prey. Moreover, the positivity did not differ significantly between the cantons of Grisons (72 %; 31/43), Valais (74 %; 14/19), and the other cantons (Bern, Ticino, Vaud, Glarus, Schwyz, Fribourg, Obwalden, and St. Gallen: 84 %; 21/25). This lack of small-scale geographic differences aligns well with the large territories and long-distance migrations of wolves. Most of the samples originated from the Alpine arch within Switzerland, corresponding with the main distribution of wolf packs in Switzerland (KORA – *Raubtierökologie und Wildtiermanagement*, https://www.kora.ch/de).

Coprological identification of *Sarcocystis* species in DHs is not feasible, as the oocysts/sporocysts shed in feces are morphologically indistinguishable. To achieve species-level resolution, we employed *18S* PCR as an initial screening method (Table 3). Two samples which contained oocysts/sporocysts, resulted negative in the screening PCR. This could be due to a lower amount of oocysts/sporocysts rendering in insufficient DNA. Monoinfections were infrequent, occurring only in 16 % (9/55) of the samples, involving *S. tenella* (6/9) and *S. arieticanis* (3/9). Mixed infections were detected in 84 % (46/55) of the cases. This high rate of mixed infections could reflect the prolonged period of oocysts/sporocysts shedding, lack of cross immunity, and the likelihood of concurrent infection with multiple *Sarcocystis* species, as its also known in other DHs (Juozaitytė-Ngugu et al., 2021). Even samples identified as monoinfections could harbor additional *Sarcocystis* spp., as overrepresentation of a single species could also result in high-quality chromatograms in Sanger sequencing. The *18S* rRNA gene has been frequently used for molecular characterization of *Sarcocystis* spp., but it offers limited discriminatory power between closely related *Sarcocystis* species, such as *S. tenella/S. capracanis*, *S. arieticanis/S. hircicanis*, and *S. linearis/S. taeniata*. To enhance taxonomic resolution, we applied *COI* PCR to samples with mixed infections (Table 4). This gene is less conserved than the *18S* rRNA gene with an intraspecific variability up to 2.5–3 % (which can overlap with the interspecific variability range of 2.5–5 %). The primer SR9, which is particularly effective for detecting *Sarcocystis* species that infect ruminant hosts (Gjerde, 2013), is relevant in this context, as ruminants are frequent prey of the predator species under investigation (Schneider et al., 2025). Using *COI* PCR, we obtained consensus sequences and short fragment sequences with high identity to defined *Sarcocystis* species in 48 % (21/44) of the mixed infections, identifying *S. capreolicanis* (9/21)*, S. linearis* (7/21)*, S. arieticanis* (3/21)*, S. gracilis* (1/21), and *S. cervicanis* (1/21)*.* Since this last sequence was too short and could not be confirmed by other methods, it was not further considered as a properly identified species in wolves as DHs. However, in 52 % (23/44) of the mixed infections we could not determine the *Sarcocystis* spp. present. Additionally, real-time PCR revealed the prevalence of *S. cruzi* in 2 % (1/46) of all mixed infection samples. Overall, 44 % (24/55) of all samples harbored *Sarcocystis* species, which could not be confidently resolved to species-level, caused by short fragments (no alignment possible) or double/superimposed peaks in conventional PCR-sequencing methods.

To address this limitation, we analyzed five representative samples with mixed infections using a third-generation sequencing technology (PacBio). Although alternative approaches were available, we selected this method for several practical and analytical reasons. Cloning, as described by Basso et al. (2020) for example, would have required sequencing of a predefined number of clones, which is impractical and of limited sensitivity, given the potential presence of up to ten *Sarcocystis* species per sample (Lesniak et al., 2017, 2018). Short-read sequencing platforms such as Illumina, as demonstrated by others (Lesniak et al., 2017, 2018; Moniot et al., 2025), could also have been employed. However, given the relatively long target regions, we opted for long-read sequencing to ensure complete coverage. This decision was further supported by the anticipated effectiveness of long-read amplicon sequencing in detecting and resolving species diversity in mixed and heterogeneous samples (Callahan et al., 2019). Additionally, PacBio long-read amplicon sequencing is known for its exceptionally low error rate – often lower than those associated with conventional Illumina sequencing (Callahan et al., 2019). This approach required the development of novel bioinformatics pipelines. Due to the lack of established reference databases for *18S* rRNA, we adapted methodologies commonly used for bacterial *16S* rRNA analysis, using the DADA2 tool as a framework (Callahan et al., 2019). This process generated a large volume of raw sequence data. From this dataset, we identified 23 ASVs for *18S* (Table 5) and more than 170 ASVs clustered into 18 OTUs for *COI* (Table 6), which already showed the higher variability rate of the last target gene. Species-level assignment was verified based on BLAST identity thresholds (*18S*: >99 %, *COI*: >96 %), supported by phylogenetic analysis. As with conventional molecular methods, it was not possible to assign all *18S* ASVs to a specific species with high confidence. Therefore, we also trimmed the sequences to the shorter fragment – corresponding to the target used in our conventional *18S* screening PCR – and compared the results using BLAST and phylogenetic placement. Nevertheless, the ASVs labeled as “like” were either with higher identity to a given species by BLAST or were within the same phylogenetic branch as that species. We subsequently realized that initially using only the short fragment of *18S* might be a better option as it includes the hypervariable region IV and it is more specific for *Sarcocystis* spp. than for other organisms such as fungi etc., which were also amplified using the *18S* (*full-length*) PCR (data not shown). In fact, fewer *18S* than *COI* reads could be assigned to *Sarcocystis* spp. (∼5 % versus ∼25 %, respectively). This indicates a higher proportion of unspecific amplicons in the PCR targeting the complete *18S* gene. Nevertheless, when both targets were considered, each sample harbored at least three known *Sarcocystis* species, with up to six species detected in a single host, confirming high levels of co-infection. Notably, three known species – *S. capracanis, S. venatoria*, and *S. iberica* – were not detected using conventional PCR-sequencing methods, highlighting the enhanced resolution of NGS in characterizing mixed infections. One ASV from *18S* full-length PCR (**PX046103**) and three OTUs from *COI* PCR (**PX000720**, **PX000721**, **PX000722**) showed low BLAST identity and were positioned in relation with two or more species in the phylogenetic tree. These sequences could either represent a not yet described *Sarcocystis* species or a potential recombination of closely related species in the intestine of a wolf. However, this hypothesis deserves further research. Additionally, the detection of one ASV from *18S* full-length PCR sequencing (**PX046104**), which was isolated from all the other reference sequences in the phylogenetic tree, may represent a novel *Sarcocystis* species or at least a species from which no sequence is reported in the GenBank. When comparing NGS performance using *18S* and *COI* targets, greater resolution was achieved with *COI*. Specifically, *S. capreolicanis, S. gracilis, S. iberica*, and *S. venatoria* could not be reliably distinguished using the *18S* target. Across all diagnostic methods combined, we identified a total of nine known *Sarcocystis* species: *S. tenella, S. arieticanis, S. capreolicanis, S. linearis, S. gracilis, S. cruzi, S. capracanis, S. iberica,* and *S. venatoria*. These may represent a subset of the species present, given that the *COI* primer combination used preferentially amplifies *Sarcocystis* spp. with ruminants as IHs (Gjerde, 2013).

The wolf had already been confirmed as DH for *S. cruzi*, as well as for *S. mehlhorni*, and *S. wenzeli* (Gupta et al., 2024b). Identification of *S. wenzeli* was based on high similarity in the *18S* rRNA and *COI* gene sequences, which provide limited resolution for distinguishing closely related avian *Sarcocystis* species (Gondim et al., 2021). However, it should be considered as a *S. wenzeli*-like species, since the more informative *ITS1* region yielded only 91 % similarity. Based on molecular analyses of fecal samples, the wolf has previously been proposed as DH for several additional *Sarcocystis* species (Lesniak et al., 2017, 2018). However, these two studies did not include coprological examinations for the presence of oocysts/sporocysts in feces. Therefore, it cannot be ruled out that the detected DNA originated from recently ingested prey, implying that the wolf should only be considered as potential DH for these species. In contrast, the present study confirms the wolf as natural DH for five of these species (*S. tenella, S. arieticanis, S. capreolicanis, S. gracilis, S. capracanis*)*.* Since we used concentrated material from sedimentation or flotation for the DNA extraction, the probability of amplifying free DNA not derived from oocysts/sporocysts was considered extremely low. Importantly, three *Sarcocystis* species – *S. linearis, S. iberica,* and *S. venatoria* – were reported for the first time in Eurasian wolves, establishing this predator as DH for these species with roe deer, red deer, sika deer and moose as HIs (Table 7). These species have been relatively recently described from roe deer (*S. linearis*) and red deer (*S. Iberica* and *S. venatoria*) and from which the DH were initially suspected to be canids based on their phylogenetic position (Gjerde et al., 2017a, 2017b). Recently, these species were found in racoon dogs (*Nyctereutes procyonoides*) as DH (Prakas et al., 2025). Interestingly, *S. linearis* was detected in all five wolf samples, suggesting a frequent wolf predation over roe deer (and other cervids), and in turn, positioning the wolf as a frequent and probably major natural DH of this species. These findings highlight the ecological role of wolves in the transmission of diverse *Sarcocystis* species and demonstrate the importance of NGS in resolving mixed infections.

**Table 7:** *Sarcocystis* spp. reported for the first time in Eurasian wolves and the known corresponding IHs.

*Sarcocystis* species	Corresponding IHs
*S. linearis*	Roe deer (*Capreolus capreolus*)^a^
	Red deer (*Cervus elaphus*)^b^
	Moose (*Alces alces*)^c^
	Sika deer (*Cervus nippon*)^d^
*S. iberica*	Sika deer (*Cervus nippon*)^d^
	Red deer (*Cervus elaphus*)^b^
*S. venatoria*	Red deer (*Cervus elaphus*)^b^

a (Gjerde et al., 2017a).

b (Gjerde et al., 2017b).

c (Prakas et al., 2019).

d (Prakas et al., 2023).

The detection of these *Sarcocystis* species is not unexpected, as most have been reported previously in other canid hosts (Dubey et al., 2016; Basso et al., 2020; Prakas et al., 2025). For each *Sarcocystis* species identified in this study, at least one IH is known that plausibly contributes to the wolf's diet in Switzerland (mainly red deer, roe deer, chamois, and occasionally livestock) (KORA – *Raubtierökologie und Wildtiermanagement*, https://www.kora.ch/de). Although infections with *Sarcocystis* species are generally subclinical in wolves, they can cause significant pathological lesions in their IHs, such as eosinophilic myositis/fasciitis in red deer (Basso et al., 2020), cattle (Rubiola et al., 2024), and other host species. Such changes render affected meat unsuitable for human consumption. Importantly, even in the absence of visible changes, the ingestion of meat containing a high number of sarcocysts has been associated with gastrointestinal symptoms (vomitus/diarrhea) in humans, posing a risk to food safety (Ota et al., 2019; Vélez et al., 2021). Further complementary studies on potential IHs, particularly prey species, will enhance understanding of host-parasite dynamics, supplement knowledge of life cycles, and provide insights into the variability of gene targets and markers used for *Sarcocystis* spp. species identification.

## 5 Conclusion

Across all diagnostic methods combined, we identified a total of nine known *Sarcocystis* species: *S. tenella, S. arieticanis, S. capreolicanis, S. linearis, S. gracilis, S. cruzi, S. capracanis, S. iberica,* and *S. venatoria*. These findings confirm the Eurasian wolf as natural DH for multiple *Sarcocystis* species for the first time using both coprological and molecular methods, including *S. linearis*, *S. iberica*, and *S. venatoria*. NGS proved essential for resolving mixed infections. This work contributes to a deeper understanding of epidemiology, predator-prey interactions, and diagnostic challenges in wildlife parasitology. Further complementary studies on prey species will enhance understanding of host-parasite dynamics.

## CRediT authorship contribution statement

**Sinah Lückner:** Writing – original draft, Project administration, Investigation, Formal analysis, Data curation. **Gastón Moré:** Writing – review & editing, Supervision, Methodology, Formal analysis, Conceptualization. **Iris Marti:** Writing – review & editing, Resources. **Caroline F. Frey:** Writing – review & editing, Validation, Funding acquisition. **Javier E. Fernandez:** Writing – review & editing, Visualization, Software, Resources, Formal analysis. **Chahrazed Belhout:** Writing – review & editing, Visualization, Software, Resources, Investigation, Data curation. **Walter Basso:** Writing – review & editing, Supervision, Methodology, Funding acquisition, Conceptualization.

## Ethical statement

No wolves were killed specifically for the purposes of this study.

## Funding

This work was supported by internal fundings from the Institute of Parasitology, 10.13039/100009068University of Bern.

## Declaration of competing interest

All the authors are free from conflict of interests which could potentially bias the present study.
